# Effect of pH on the Corrosion and Repassivation Behavior of TA2 in Simulated Seawater

**DOI:** 10.3390/ma14226764

**Published:** 2021-11-10

**Authors:** Yingxiao Zhang, Tingting Yan, Lin Fan, Zhiyong Liu, Longfei Song, Xiaogang Li

**Affiliations:** 1 Corrosion and Protection Center, Key Laboratory for Corrosion and Protection (MOE), University of Science and Technology Beijing, Beijing 100083, China; zhangyingxiao0515@163.com (Y.Z.); zyxzyy2008@163.com (T.Y.); flynnfan@163.com (L.F.); songlongfei@gzhu.edu.cn (L.S.); 2 National Materials Corrosion and Protection Data Center, University of Science and Technology Beijing, Beijing 100083, China; 3 State Key Laboratory for Marine Corrosion and Protection, Luoyang Ship Material Research Institute (LSMRI), Qingdao 266101, China

**Keywords:** TA2, corrosion behavior, repassivation behavior, simulated seawater environment

## Abstract

The effect of pH on the corrosion and repassivation behavior of TA2 in simulated seawater was studied using electrochemical tests, immersion experiments, and surface morphology topology analysis. The results show that E_corr_ and R_f_ increased while i_pass_ and weight loss rate decreased as the pH of simulated seawater increased. The TA2 passive film was determined to be mainly composed of a large amount of TiO_2_ and a small amount of TiO. The repassivation function of TA2 can be expressed as *E* = −0.1375 + 0.0532ln(*t* − 1.241) for a simulated seawater pH of 8.2. The parameter b, which represents the slope of the potential–time curve during the friction electrode test, was used to evaluate the repassivation behavior of TA2. The increase in pH value was observed to promote the repassivation speed of the passive film, which is beneficial to the corrosion resistance of TA2.

## 1. Introduction

Corrosion is a major problem and bottleneck encountered in the marine environment, especially for marine engineering structures and military installations such as offshore platforms and warships. Therefore, the development of high-end corrosion resistant materials and promotion of their application is of great importance in the above-mentioned fields [1,2,3]. The excellent corrosion resistance of TA2 makes it have broad application prospects, such as biomedical [4], desalination [5], and marine facilities [6,7]. The surface of titanium can quickly form a dense, well-covered and difficult-to-dissolve passive film. This passive film mechanically separates the metal from seawater, greatly lowering the metal dissolution rate, and has excellent self-repairing ability [8,9]. The corrosion resistance of titanium depends on the integrity of the passive film on the surface and its self-repairing ability after rupture. Cui et al. [10] studied the corrosion behavior of TA2 in simulated desulfurized flue gas condensates and found that the passive film broke and Ti dissolved. Once the passive film is destroyed in severe marine environments, such as in deep sea or where high-level stress is encountered, the titanium may quickly corrode and fail [11].

The performance of passive films is affected by many factors, such as temperature, pH value, and dissolved oxygen content in the marine environment. Among them, the influence of pH value is of wide concern. Gurrappa et al. [12] found that when IMI-834 titanium was in a 0.5 M H_2_SO_4_ solution, its corrosion potential value was more positive and its corrosion current density value was lower than when it was in a 3.5% NaCl solution. Li et al. [13] found that the passivity of 316 L stainless steel was enhanced with increasing pH value. Gou et al. [14] prepared a titanium-containing conversion coating on an AZ91 alloy and found that the increase in pH value promoted a decrease in the coating thickness and crack number, consequently improving the corrosion resistance. These results suggest the possibility that the seawater pH affects the corrosion behavior of passive metals such as titanium alloys. Hence, to investigate the corrosion behavior of TA2 in seawater, comprehensive research under different pH values is necessary.

The repassivation characteristics are a very important property of passive metals. If the passive film is destroyed, the metal substrate will come directly into contact with the corrosive environment, and dissolution is likely. If the passive film can be quickly repaired at this time, the metal substrate will be less affected by corrosion. If not, the metal substrate will continuously dissolve, and severe local or uniform corrosion will occur, which will cause significant losses. Therefore, studying the repassivation kinetics of metals is of great significance for the service safety of equipment and facilities. In this regard, Scully et al. [15,16] found that film formation and anodic dissolution of metals kinetically led to the initiation of pitting, which might depend on the repassivation rate. Burstein and Marshall et al. [17] studied the repassivation behavior of 304 L stainless steel in 1.0 M KOH solution and found that an increase of the repassivation rate improves the corrosion resistance. Domene et al. [18] studied the repassivation kinetics of titanium in LiBr solution under cavitation corrosion, which conforms to the relationship *i*(*t*) = A*t*^−n^, where *i*(*t*) is the anodic current density, A is a constant, *t* is time, and n is the repassivation index. Sakairi et al. [19] studied the effect of the applied potential of titanium under artificial saliva and the effect of F^−^ on the kinetics of titanium repassivation. Therefore, studying the repassivation behavior is essential to evaluating the corrosion resistance of passive metals.

At present, there is little research and data on the regularity of titanium corrosion behavior in marine environments. Studying the influence of different factors on the passive film of titanium in the marine environment and damage repair mechanism of titanium can provide assurance for safety issues in marine engineering.

In this study, electrochemical tests, immersion experiments, and morphology analysis were used to investigate the effect of pH value on the corrosion behavior of TA2, and the friction electrode method was used to study the repassivation behavior. A convenient and quantitative analysis method is proposed for evaluating the repassivation behavior of TA2. This work aims to fill the lack of corrosion data in the literature, and the results help to ensure the safety of long-term service equipment in the marine environment.

## 2. Experimental

### 2.1. Materials and Specimens

The titanium TA2 used in this study had the following chemical composition in mass percentage (wt.%): 0.31 Fe, 0.20 O, 0.14 Si, 0.11 C, and Ti balance. Samples were sectioned into pieces with dimensions of 10 mm × 10 mm × 3 mm (for electrochemical tests) and 50 mm × 20 mm × 3 mm (for immersion tests and XPS tests). Samples to be used in electrochemical tests were embedded in epoxy resin to leave a 1 cm^2^ working area, or this step was otherwise skipped. All specimens were grounded with silicon paper to 2000 grit, and then ethanol and deionized water were used to clean specimen surfaces in ultrasonic cleaning machine before each experiment.

### 2.2. Electrochemical Analysis

The electrochemical measurements were conducted on a Princeton Applied Research PARSTAT 3F electrochemical workstation (Oak Ridge, TN, USA). A three-electrode cell system was employed: the TA2 sample is the working electrode (WE), a platinum sheet is the counter electrode (CE), and a saturated calomel electrode (SCE) is used as the reference electrode (RE). Before each measurement, the open circuit potential (OCP) was recorded for 1 h to obtain the stable state. Electrochemical impedance spectroscopy (EIS) data were recorded from 100 kHz to 10 mHz with an AC amplitude of 10 mV and were fitted using ZSimpWin software (ZSimpWin 3.50, Ann Arbor, MI, USA). The potentiodynamic polarization curves were recorded from −500 mV (vs. OCP) to 4 V (vs. SCE) at a sweep rate of 1 mV/s. The capacitance measurements were performed from 3 V (vs. SCE) to −1 V (vs. SCE) at a fixed frequency of 1 kHz using a 10 mV AC signal and a step rate of 50 mV/s. The cyclic voltammetry was conducted from −1.75 V (vs. SCE) to 0.5 V (vs. SCE) at scan rates of 50, 100, 200, 400, 800, and 1000 mV/s.

The solution used in this work was simulated seawater, the composition (g/L) of which was 24.53 NaCl, 5.2 MgCl_2_, 4.09 Na_2_SO_4_, 1.16 CaCl_2_, 0.695 KCl, 0.201 NaHCO_3_, 0.027 H_3_BO_3_, 0.0025 SrCl_2_, and 0.003 NaF. The seawater pH did not maintain at 8.2, which is an average level of seawater pH values. The pH of the local environment (including but not limited to the solution in crevices or mine) in seawater may deviate from this level. Therefore, all the measurements were conducted at different pH values to obtain variables and study the effect. The seawater pH was adjusted to 1, 4, 7, and 10 by 0.1 mol/L HCl and NaOH. All experiments were performed at 25 °C and 0.1 MPa.

### 2.3. Immersion Tests

The corrosion resistance was evaluated using weight loss experiments in simulated seawater with different pH values (same as the solution in Section 2.2) for 90 days. Fishing line was used to make sure that samples were completely immersed in the solution without contact. Samples were cleaned by ethanol and deionized water, and dried before weighing by an electronic analytical balance (with 0.0001 g precision). Three sets of parallel samples were set up for the experiments corresponding to different pH. Weight loss rate was calculated by the following equation:

(1) $$V=\left(W_{1}-W_{2}\right)/\left(S\cdot T\right)$$

where *W*_1_ is the weight before the immersion test, *W*_2_ is the weight after the immersion test, *S* is the working area of the sample, and *T* is the duration of the immersion test.

### 2.4. Morphology Analysis

To study the effect of pH on the passivation, the sample surface morphology was observed using a FEI Quanta 250 scanning electron microscope (SEM, Thermo Fisher Scientific, Waltham, MA, USA) after immersion tests. X-ray photoelectron spectroscopy (XPS, Thermo Fisher Scientific, Waltham, MA, USA) was also used to analyze the surface composition of the specimen. A Thermo ESCALAB 250 Xi device was employed to conduct XPS measurement (Thermo Fisher Scientific, Waltham, MA, USA). The device was equipped with an Al-Ka (1486.6 eV) as an X-ray source. The binding energy was calibrated by referencing the C1s peak (284.8 eV). After measurements, the XPS spectra were fitted by XPS-Peak 4.1 software (freeware written by Raymond Kwok, Hong Kong, China).

### 2.5. Friction Electrode Test

To study the effect of pH on the repassivation behavior of TA2, an abrading electrode technique was employed to conduct friction electrode tests. The embedded electrochemical samples (as working electrode) were mechanically abraded by a 1500 grit SiC disk; meanwhile, an electrochemical workstation was connected to samples to record potential data. The data were recorded at a rate of 10 points/s and the test lasted 250 s. The tests were performed at 25 °C and 0.1 MPa in simulated seawater. Before the tests, samples were grounded by SiC paper to 2000 grit and immersed in simulated seawater for 30 min to ensure the OCP was stable.

## 3. Results

### 3.1. Electrochemical Characteristics

**Figure 1:**
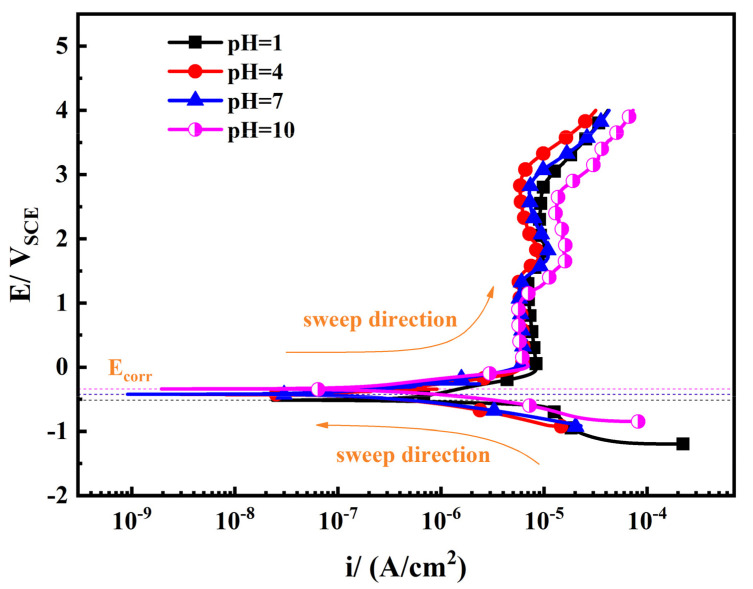
Potentiodynamic polarization curves of TA2 in simulated seawater with different pH values.

To investigative the kinetics of the corrosion process, the potentiodynamic polarization curves of TA2 in simulated seawater with different pH values were determined and are shown in Figure 1. It can be seen that anodic current density of TA2 levels off at 0 V (vs. SCE), suggesting obvious active-passive behavior. TA2 maintains extremely low current density under different pH conditions. The passivation zone starts at about 0 V (vs. SCE), and the breakdown potential was not observed during the test. This range is considerably wide compared with other passive metals such as 316 L stainless steel [20], 2205 duplex stainless steel [21], and Ni(Fe, Al)-maraging steel [22], and was due to the protection of the passive film on the metal substrate surface. As the potential increases, the inflection points of the passivation zone of the polarization curve under different pH conditions can be observed. The passivation zone of each curve is divided into two regions at about 1.5 V (vs. SCE). This behavior can be attributed to the changes in the composition and structure of the passive film [23,24,25,26].

**Figure 2:**
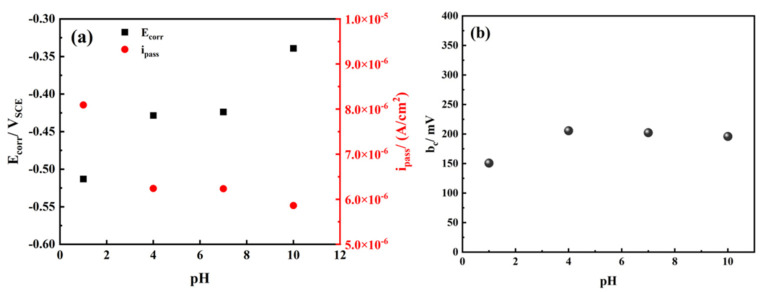
Variations in (**a**) corrosion potential *E*_corr_ and passive current density *i*_pass_, (**b**) cathodic Tafel slope *b*_c_ obtained from potentiodynamic polarization curves of TA2 in simulated seawater with different pH values.

Accordingly, variations in corrosion potential (*E*_corr_) and passive current density (*i*_pass_) obtained from potentiodynamic polarization curves of TA2 in simulated seawater with different pH values are shown in Table 1 and Figure 2a. Considering the passive behavior of TA2 shown on the anodic branch of potentiodynamic polarization curves, the passive current density instead of I_corr_ was used to analyze the electrochemical reaction speed of TA2. In the natural state, TA2 could maintain passive state suggesting the anode was polarized to the passive zone. Therefore, the passive current density obtained from the passive zone was more suitable to analyze the potentiodynamic polarization curves than I_corr_ calculated by the intersection of Tafel curves. It can be seen in Figure 2a that *E*_corr_ increases with the increasing pH, suggesting the tendency to corrode weakens. When the pH is between 4 and 10, the *i*_pass_ values are basically the same. When in a strong acid environment (pH = 1), the passive current density is slightly higher than that of other conditions, suggesting that the corrosion resistance is weaker. The cathodic Tafel slope (*b*_c_) is calculated and shown in Figure 2b. When the pH is between 4 and 10, the *b*_c_ of TA2 in the simulated seawater is nearly invariant and independent of pH values at about 200 mV. This result is consistent with the findings of other studies [26,27]. When the pH is 1, the *b*_c_ of TA2 obviously decreases and has a value of 150 mV. This indicates that the cathodic reaction of TA2 is easier to proceed in a strongly acidic condition.

**Figure 3:**
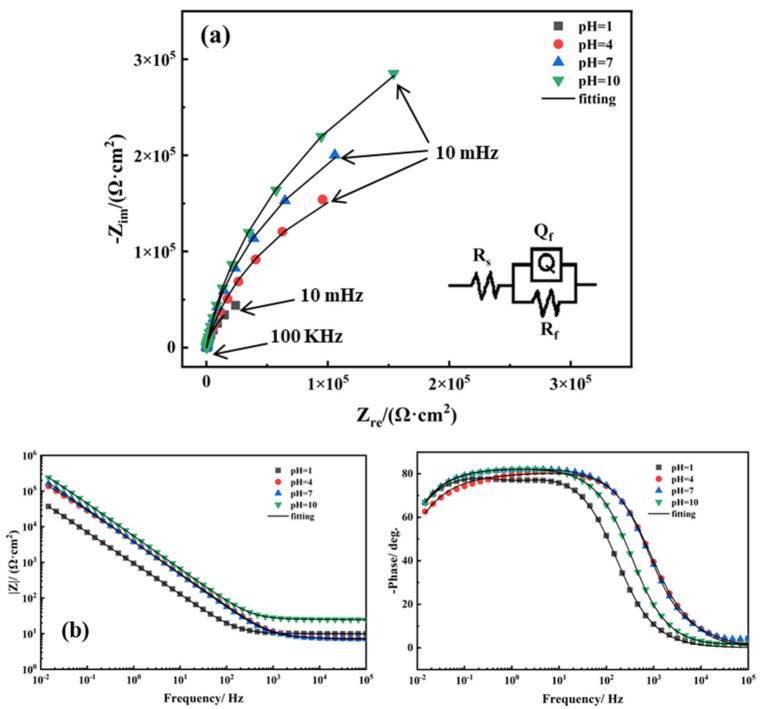
EIS curves and equivalent circuit of TA2 in simulated seawater with different pH values: (**a**) Nyquist plots, (**b**) Bode diagrams.

**Figure 4:**
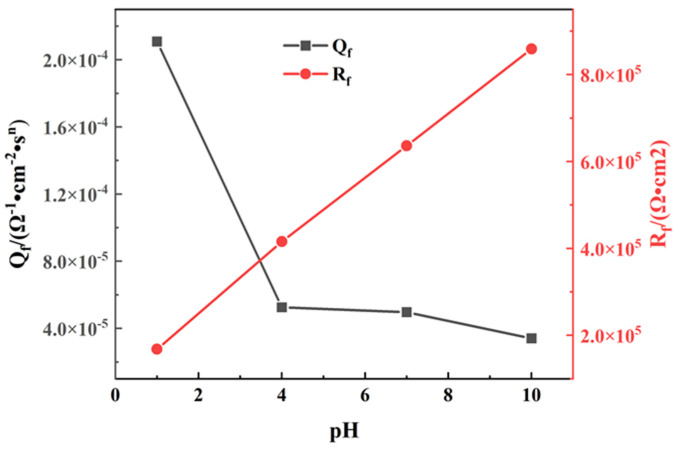
Variations in *Q*_f_ and *R*_f_ as a function of pH.

Figure 3 shows the electrochemical impedance spectra (EIS) and equivalent circuit of TA2 in simulated seawater environments with different pH values. The impedance spectra of TA2 characterized by an unfinished semicircular arc, is typical of passive system impedance. There is only one capacitive reactance arc, suggesting only one time constant without diffusion resistance. The moduli of impedance show a linear correlation with a slope of approximately −1, and the phase angles are at a range of 60–80° at the low and middle frequency area, suggesting that in terms of its characteristics, the film is mainly capacitive. As the pH value increases, the radius of capacitive reactance arcs increases significantly, and the impedance value increases. As the pH value decreases, the phase angle of the mid-low frequency shows a decreasing trend. Contact between the passive film and the metal substrate surface was maintained due to the high speed of oxide formation [28]. Therefore, as a result of the intact layer at the electrode–electrolyte interface, no charge transfer process occurred, and the simple Randles circuit was used to fit the EIS curves, which was consistent with those of other studies [29,30]. In the equivalent circuit model, *Q* is a constant phase element (CPE) resulting from the different degrees of heterogeneity at the electrode surface [31], whose impedance obeyed the following relationship [7]: *Z*_CPE_ = C(j2π*f*)*^n^*^−1^, where C is the capacitive constant, j is the imaginary unit, *f* is the frequency, and *n* is a CPE power that is between 0 and 1. *Q* is the pure capacitance when *n* = 1, and *Q* is the pure resistance when *n* *=* 0 [32]. *R*_s_ is the solution resistance, *Q*_f_ represents the CPE of the passive film, and *R*_f_ is the resistance of film. These parameters of the circuit elements were calculated and are listed in Table 2. Figure 4 shows variations in *Q*_f_ and *R*_f_ as a function of pH. As seen, *Q*_f_ decreases when the pH increases, and *R*_f_ shows an approximately linear increasing trend. *Q*_f_ reflects the dielectric behavior of the passive film. The increase of *Q*_f_ means the increase of micro-flaws in the passive film [33,34]. The decrease trend of *Q*_f_ when pH values vary from 1 to 10 indicates that increase in pH value improves the passive film. In addition, *n* reflects roughness of the electrode [35]. As the pH value increases, the value of *n* slightly increases, suggesting that scattering effect is weakened and roughness of the electrode surface increases. The film resistance steadily increases from 1.681 × 10^5^ Ω·cm^2^ to 8.594 × 10^5^ Ω·cm^2^ when the pH increases from 1 to 10. According to Cui et al. [36], the *C*_eff_ could be calculated by the following equation: *C*_eff_ = Y_0_^1/*n*^ *R*_s_ ^(1−*n*)/*n*^, where Y_0_ is the magnitude of CPE (*Q*_f_), *R*_s_ is the solution resistance, and *n* is the CPE power. The values of the circuit elements are listed in Table 2. As seen, the *C*_eff_ under pH condition of 1 is a maximum and the *C*_eff_ under pH condition of 10 is a minimum. These results indicate that the increase in pH value is beneficial for improving TA2 corrosion resistance.

**Figure 5:**
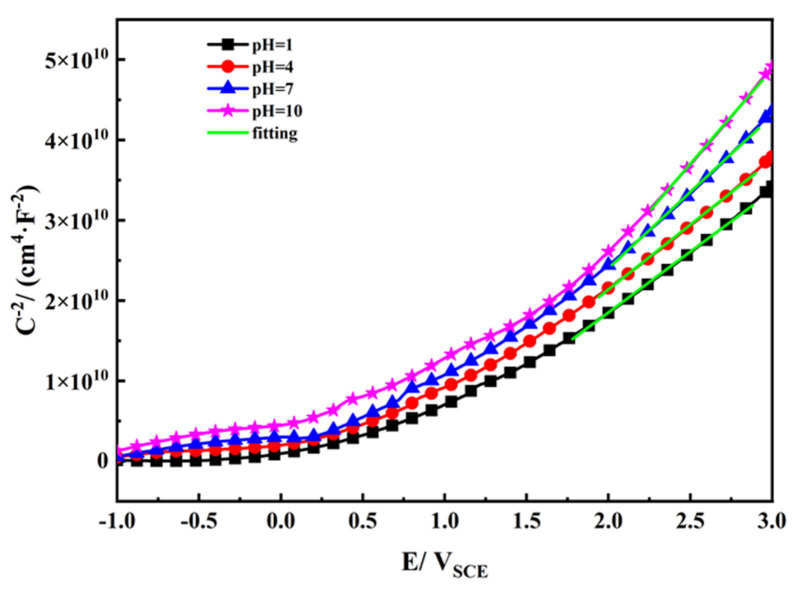
Mott–Schottky plots of TA2 in simulated seawater with different pH.

In order to study the semiconductor characteristics of the passive film, Mott–Schottky plots of TA2 in simulated seawater with different pH values were determined, as shown in Figure 5. When the potential is between 2 V (vs. SCE) and 3 V (vs. SCE), a linear region is found regardless of the pH value. It can be seen from the positive slope that the passive film on the surface of TA2 exhibits n-type semiconductor characteristics, and the structure of the passive film has oxygen vacancies or metal cation defects [37,38]. The relationship between the space-charge capacitance and applied potential can be expressed by the following equation [37,39]:

(2) $$\frac{1}{C^{2}}=\frac{2}{eN_{D}\varepsilon \varepsilon_{0}A^{2}}\left(E-E_{FB}-\frac{kT}{e}\right)$$

where *C* is the space-charge capacitance, e is the electric charge of the carrier, *N*_D_ is the donor carrier density, *ε* is the film relative permittivity (56) [40], *ε*_0_ is the vacuum permittivity (8.854 × 10^−14^ F/cm) [40], *E* represents the applied potential, *E*_FB_ is the flat band potential, *k* is the Boltzmann constant, and *T* is the temperature.

**Figure 6:**
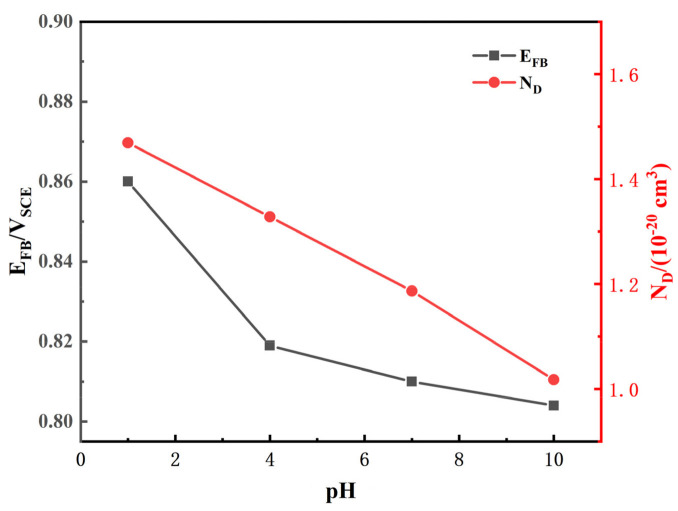
Variations in *E*_FB_ and *N*_D_ as a function of pH.

Donor carrier density (*N*_D_) and flat band potential (*E*_FB_) were calculated and are listed in Table 3. Figure 6 shows variations of *E*_FB_ and *N*_D_ as a function of pH. As seen in Figure 6, both *N*_D_ and *E*_FB_ increase with decreasing pH. *N*_D_ can be used to characterize the point defect concentration of the passive film and is related to its composition and the amount of surface charge. Thus, when the pH decreases, the passive film becomes more conductive and its corrosion resistance becomes relatively weak, which is consistent with the EIS results in Figure 3.

**Figure 7:**
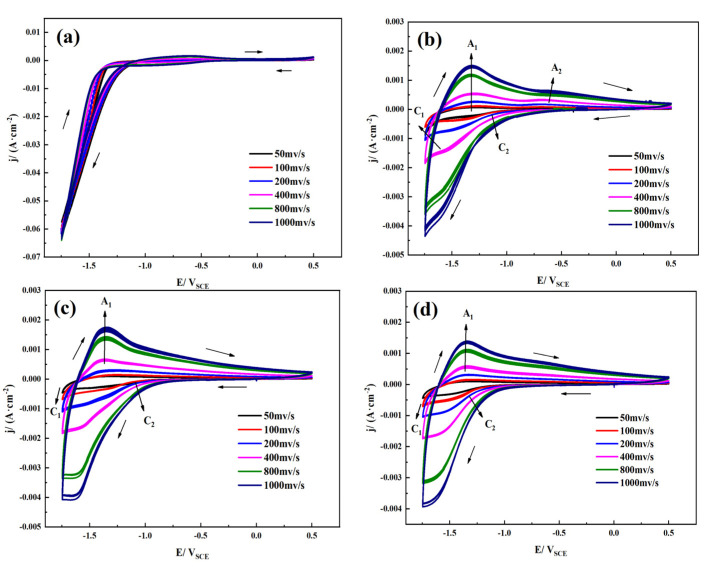
Cyclic voltammetry plots of TA2 in simulated seawater with different pH values using different scan rates: (**a**) pH = 1, (**b**) pH = 4, (**c**) pH = 7, and (**d**) pH = 10.

Figure 7 shows the cyclic voltammetry plots of TA2 under different scanning rates and different pH values in simulated seawater. As the scanning rate increases, the current and redox peaks become larger, and the reduction peak and oxidation peak potentials shift slightly, suggesting a worsening of the oxide film stability. There is little difference in the shape of the cyclic voltammetry curves when the pH value is 4, 7, and 10. The current density of the cyclic voltammetry curve under the condition of pH = 1 is one order of magnitude higher than the current density under other conditions. The dissolution rate of the oxide film increases as the current density increases, and there is more serious corrosion of TA2. TA2 corrodes more severely under strongly acidic conditions (pH = 1) than other conditions. The cyclic voltammetry plots of TA2 show little difference when the pH is between 4 and 10, which is consistent with the results of the polarization curves in Figure 1. It can be seen from the cyclic voltammetry plots that the wave height of the redox peak is inconsistent, the curve shape is asymmetric, and the electrode surface reaction is completely irreversible; that is, the oxidation process of metallic titanium is irreversible. This is because the surface of titanium is active, and it is very easy to generate titanium dioxide oxide with oxygen in water or oxygen atoms deprived of water to passivate the surface of titanium and protect the metal substrate [7]. We next calibrate each characteristic peak of the cyclic voltammetry plots. Peak A1 corresponds to the formation process of the passive film, with oxidation of Ti to Ti^2+^, and peak A2 corresponds to the oxidation of Ti^3+^ to Ti^4+^. Peak A2 is not obvious because the reaction process proceeds too quickly to allow the detection of changes in current. Peak C2 corresponds to the reduction of Ti^4+^ to Ti^3+^, and peak C1 corresponds to the reduction of Ti^2+^ to Ti. This shows that TiO, Ti_2_O_3_, and TiO_2_ are sequentially formed in the passive film.

### 3.2. Immersion Tests

**Figure 8:**
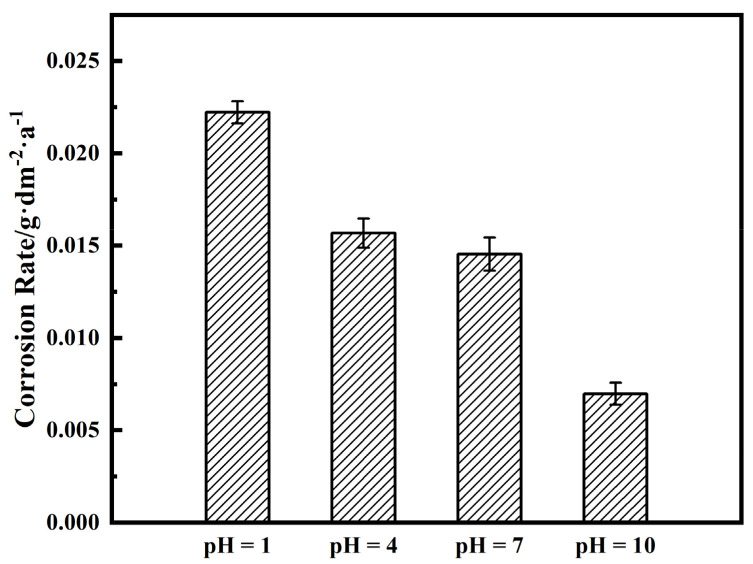
Corrosion rate of TA2 in simulated seawater with different pH values.

Figure 8 shows the corrosion rate of TA2 in simulated seawater with different pH values, which is extremely low. With the increase in pH, the corrosion rate of TA2 generally shows a decreasing trend, which is consistent with the results of *i*_pass_ in Figure 2 and *R*_f_ in Figure 4. TA2 exhibited similar corrosion rates in seawater with pH values of 4 and 7. The corrosion rate of TA2 was the lowest at pH 10 and had a value of 0.006968 g·dm^−2^·a^−1^. When the pH is at 1, the corrosion rate of TA2 was the highest, with a value of 0.02222 g·dm^−2^·a^−1^. This shows that in the case of higher H^+^ concentration, the protective performance of the passive film of TA2 was weaker.

### 3.3. Morphology Observation and Composition Analysis

**Figure 9:**
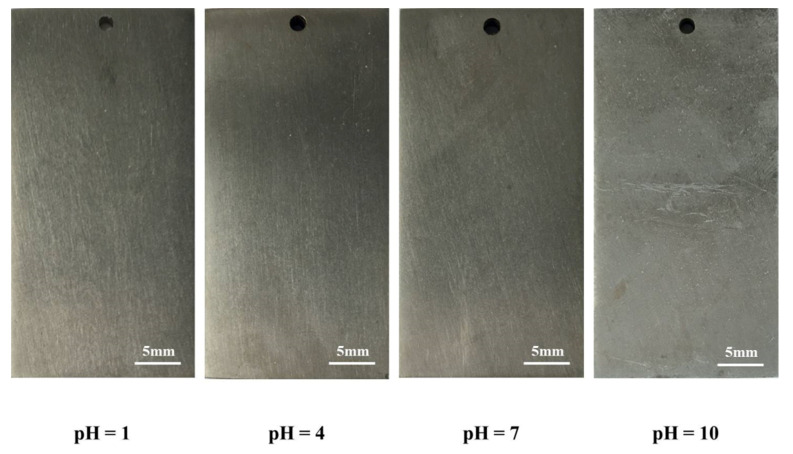
Macroscopic morphology of TA2 in simulated seawater solution with different pH values.

Figure 9 shows the macroscopic morphology of TA2 in simulated seawater solution at different pH values. It can be seen that the passive film of TA2 remained in a stable state even in a strongly acidic environment with a pH of 1, thus protecting the base metal. The surface of the material appears shiny with a metallic luster, as when it was just immersed in the solution, and no obvious corrosion product is observed. Under the condition of pH = 10, the color of pH = 10 sample surface was gray-like with weak luster and those of others were silver-like with obvious luster, suggesting that precipitation from seawater formed and was adsorbed on the metal surface.

**Figure 10:**
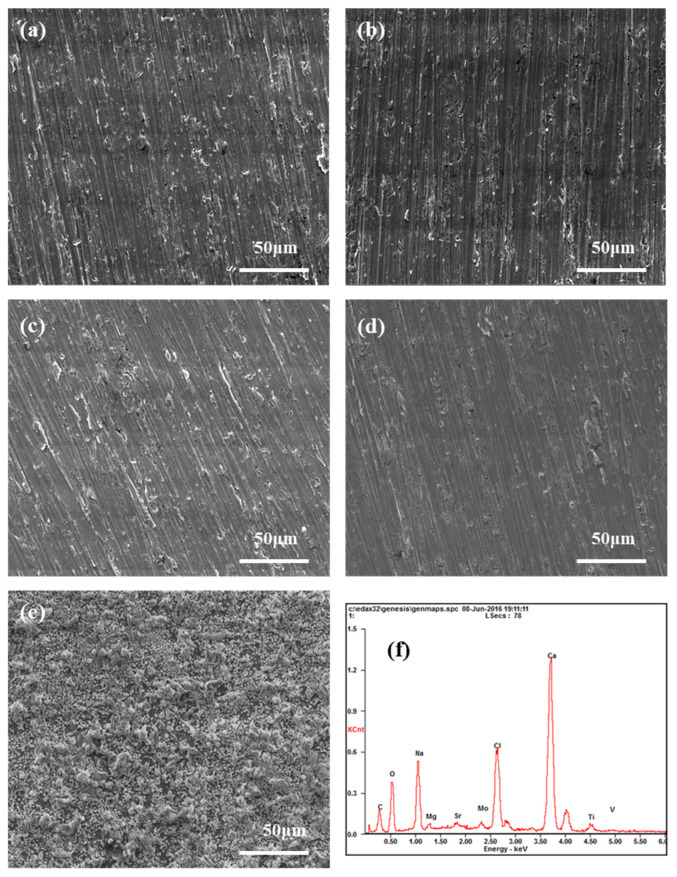
Microscopic morphology of TA2 in simulated seawater solution with different pH values: (**a**) pH = 1, (**b**) pH = 4, (**c**) pH = 7, (**d**) pH = 10 after removing the sedimentation layer, and (**e**) pH = 10 before removing the sedimentation layer. (**f**) EDS spectrum corresponding to analysis of the area indicated by the red box in (**e**).

Figure 10 shows the morphology of TA2 as observed by SEM after being immersed in the simulated seawater solution at different pH values. After three months of immersion, some scratches were observed on the surface of each pH condition, which were produced by silicon paper in the sample preparation process. Additionally, no pit was observed under different pH values, suggesting the excellent corrosion resistance of TA2. It should be noted that some black dots were likely to be wear track resulting from grinding with 2000 grit silicon paper before immersion test. Similar phenomenon could be found in morphology of other studies [41,42,43]. As shown in Figure 10e, a layer of white sedimentation product was deposited on the surface of the sample. Based on the result of EDS in Figure 10f, it was inferred that this layer of sedimentation was composed of calcium salt, sodium salt, etc. Together with the results shown in Figure 9, we surmise that this layer of white sedimentation product protected the substrate from contact with the solution and reduced the corrosion rate of metals under slightly alkaline conditions.

**Figure 11:**
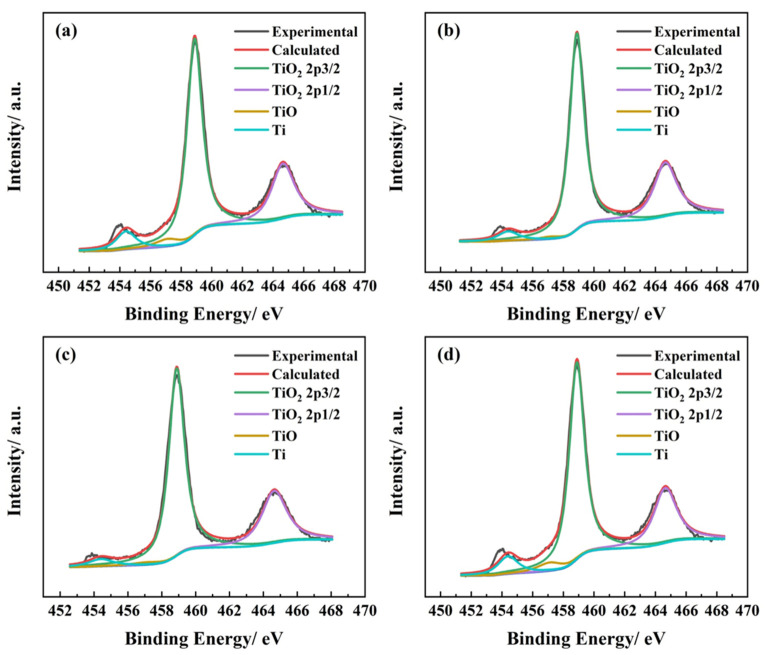
Detailed Ti2p XPS spectra of TA2 in simulated seawater with different pH values: (**a**) pH = 1, (**b**) pH = 4, (**c**) pH = 7, and (**d**) pH = 10.

The TA2 passive film components greatly affect its semiconductor properties and corrosion resistance. Figure 11 shows the XPS analysis of the passive film composition in the simulated seawater environment with different pH values. It should be noted that the layer of sedimentation under the condition of pH = 10 was removed from the sample surface without hurting the passive film before XPS measurement for convenience and quality of data. The peaks can be seen from the figure and in the XPS analysis that Ti2p3/2 (458.88 eV) and Ti2p1/2 (464.64 eV) in TiO_2_, Ti2p3/2 (457.12 eV) in TiO, and Ti2p3/2 (454.4 eV) in metallic Ti were detected. The composition percentages of TA2 passive film were calculated according to ratio of peak area in Figure 11 and are listed in Table 4. The composition of the passive film is basically the same under different conditions, composed of a large amount of TiO_2_ and a small amount of TiO. Similar results were observed by Wu et al. [43] and Ren et al. [42]. The detection of metallic Ti suggests that the passive film of the TA2 was extremely thin.

### 3.4. Repassivation Kinetics

**Figure 12:**
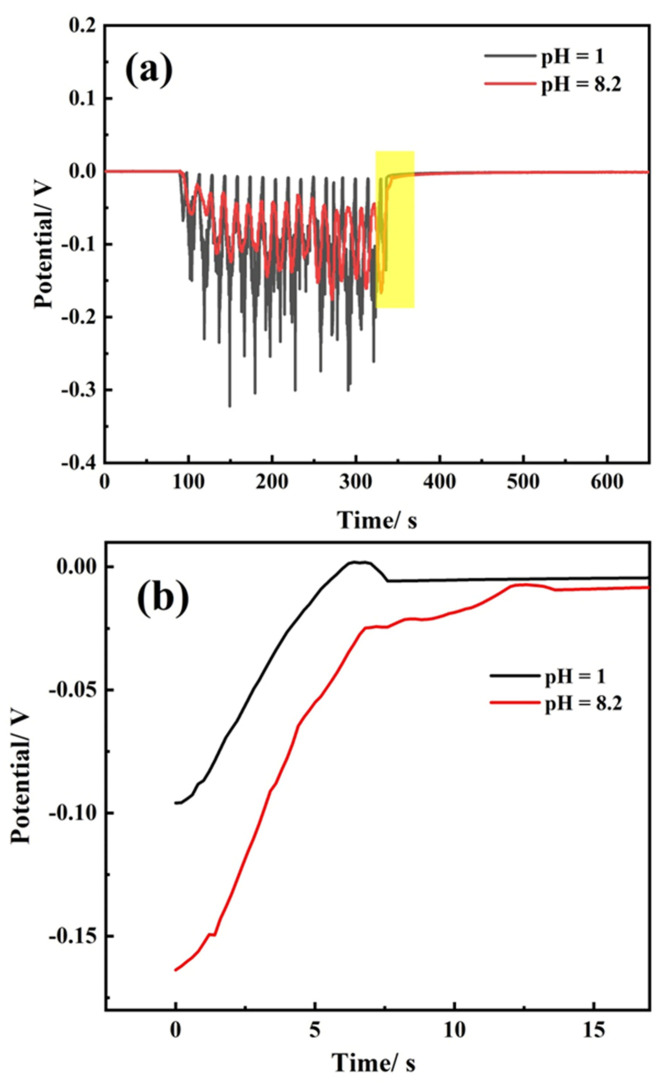
Potential–time curves friction electrode test carried out when the pH was 1 and 8.2 for (**a**) all periods of the test and (**b**) the last peak marked in (**a**).

Figure 12 shows potential–time curves obtained from the friction electrode test carried out when the pH was 1 and 8.2, where it is seen that the potential always fluctuates when the electrode surface is rubbed. Figure 12b shows details of the last peak marked in Figure 12a. As seen, the potential and time obeyed the relation of *E* = a + bln(*t* + c), where a, b, and c are constants, *E* is the potential between the working electrode and OCP, and *t* is time. The potential–time curves of the last peak were fitted, and the parameters are listed in Table 5. The repassivation function of TA2 can be expressed as *E* = −0.1375 + 0.0532ln(*t* − 1.241) when the pH of simulated seawater was 8.2. When the pH of simulated seawater was 1, the repassivation function can be expressed as *E* = −0.1228 + 0.0519ln(*t* − 1.420). The value of parameter b increased slightly as the pH value increased. The potential shifted to become more positive in the same period, suggesting that the increasing pH value promoted the repassivation of TA2.

## 4. Discussion

### 4.1. Effect of pH on TA2 Corrosion Resistance

TA2 maintained a passive state in the simulated seawater, and its electrochemical processes mainly include the process of anodic dissolution and cathodic oxygen absorption. The following reactions occur [44,45]: O_2_ + 2H_2_O + 4e^−^ → 4OH^−^, Ti + H_2_O → TiO + 2H^+^ + 2e^−^, 2TiO + H_2_O → Ti_2_O_3_ + 2H^+^ + 2e^−^, 2Ti_2_O_3_ + H_2_O → 2TiO_2_ + 2H^+^ + 2e^−^. The XPS results shown in Figure 11 indicate that the passive film composition remains basically the same under different pH conditions, composed of a large amount of TiO_2_ and a small amount of TiO. Ti_2_O_3_ was not observed, which is due to the reaction of 2Ti_2_O_3_ + H_2_O → 2TiO_2_ + 2H^+^ + 2e^−^.

The excellent corrosion resistance of TA2 can be seen from the extremely low level of i_pass_ in Figure 1 and the corrosion rate in Figure 8, and benefits from the superior resistance of the stable and tenacious passive film formed on the metal substrate surface. However, the change in pH still has effects on the passive film properties. As seen in Figure 6, *N*_D_ increases when pH decreases, which is due to the inhibition of anode reaction under high H^+^ concentrations. For n-type semiconductors, *N*_D_ is related to the amount of oxygen vacancies or metal cation defects. Thus, when the pH decreases, the passive film becomes more conductive and its corrosion resistance becomes relatively weak. This leads to a decrease in the shielding performance of the passive film, corresponding to the decrease in *R*_f_ observed in Figure 4, and the increase in *i*_pass_ observed in Figure 2. This means that the corrosion resistance of TA2 in seawater decreases with decreasing pH.

### 4.2. Effect of pH on the Repassivation Behavior of TA2

**Figure 13:**
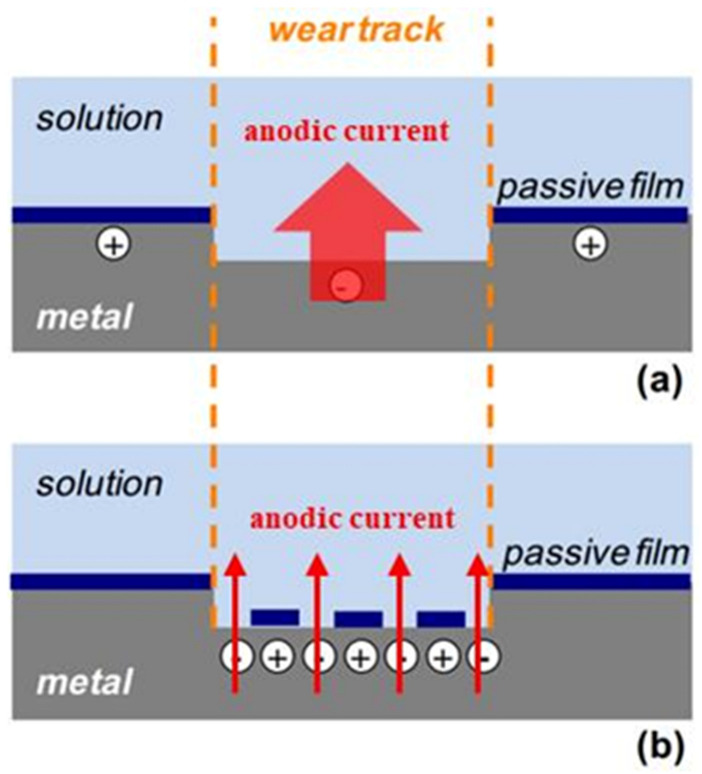
Schematic diagrams of the repassivation mechanism for (**a**) the metal substrate in direct contact with the solution when the passive film is removed by friction and (**b**) passive film growth in the initial period of repassivation.

Figure 13 shows schematic diagrams of the repassivation mechanisms in the friction electrode test. In Figure 13a, once the passive film was removed by friction, the metal substrate came directly in contact with the solution and quickly participated in electrochemical reactions, losing electrons and forming oxides, corresponding to the instantaneous decreases in potential in Figure 12a. Meanwhile, the galvanic effect can facilitate this process. As the friction electrode slides, the passive film at the wear track is removed, and the potential is negative as the anode, while the non-rubbing area is still in the passive state and has a relatively positive potential as the cathode. The galvanic cell comprises a large cathode and a small anode. In Figure 13b, some parts of the bare metal substrate preferentially repaired and formed passive film, while other parts were still bare and not repaired. In this case, the repaired part is recognized as the cathode, and the damaged part is in the activated state as the anode, forming a galvanic corrosion pair. As a result, the potential shifts positively and the current increases. In a friction cycle, the potential fails to return to the original potential. The potential of TA2 is quickly recovered at the end of the test, suggesting good repassivation properties.

According to Cho et al. [46], film grows as a place-exchange mechanism on the bare metal substrate surface, and grows as a high-field model after a monolayer forms. All currents flowing through the anode can be divided into two parts: currents for dissolution and from film formation [47]. In the initial period of repassivation, there is substantial metal substrate dissolution due to the missing protection of the passive film. This may lead to the deviation from high-field behavior. However, the formation occurs extremely quickly, and once the monolayer covers the surface, the film growth process will follow the high-field model. As solution pH increases, the film formation reaction is hindered, corresponding to the increase in *N*_D_ and decrease of *R*_f_ and repassivation speed (b in the function *E* = a + bln(*t* + c)).

## 5. Conclusions

TA2 can maintain passivation and an extremely low corrosion rate (0.006968−0.02222 g·dm^−2^·a^−1^) in simulated seawater with a pH value between 1 and 10. As the pH value decreases, the corrosion resistance of TA2 decreases. Specifically, corrosion potential and resistance of film decrease, while passive current density, capacitance of the film, micro-flaws and point defect concentration in the passive film increase. The passive film on the TA2 surface is composed of 88.11~94.31% TiO_2_, which plays an excellent protective role in corrosion resistance.

The repassivation function of TA2 could be expressed as *E* = −0.1375 + 0.0532ln(*t* − 1.241) when the pH of simulated seawater was 8.2. The parameter b, which represents the slope of potential−time curve during the friction electrode test, was used to evaluate the repassivation behavior of TA2. The increase in pH value enhances the repassivation speed of the passive film, which is beneficial to the corrosion resistance of TA2.

## Figures and Tables

**Table 1:** Polarization parameters of TA2 in simulated seawater with different pH values.

pH	1	4	7	10
*E*_corr_/mV_SCE_	−0.5131	−0.4285	−0.4239	−0.3392
I_pass_/μA·cm^−2^	8.09	6.24	6.23	5.86
*b*_c_/mV	150	205	202	196

**Table 2:** The parameters of circuit elements of TA2 in simulated seawater with different pH.

	*R*_s_/(Ω·cm^2^)	*Q*_f_/(10^−5^Ω^−1^·cm^−2^·s^n^)	*n*	*R*_f_/(10^5^ Ω·cm^2^)	*C*_eff_/(10^−5^ F·cm^−^^2^)
pH = 1	9.873	20.1089	0.88	1.681	8.61
pH = 4	7.374	5.259	0.89	4.157	1.99
pH = 7	7.295	4.969	0.91	6.362	2.27
pH = 10	24.95	3.411	0.92	8.594	1.84

**Table 3:** Fitting results of Mott–Schottky curve of TA2 in simulated seawater solution with different pH.

pH	1	4	7	10
*E*_FB_/V_SCE_	0.860	0.819	0.810	0.804
*N*_D_/(10^−20^ cm^3^)	1.469	1.328	1.187	1.018

**Table 4:** The composition of the TA2 passive film formed under different pH values.

Composition	pH = 1	pH = 4	pH = 7	pH = 10
TiO_2_	88.62%	94.28%	94.31%	88.11%
TiO	3.89%	1.17%	1.73%	4.42%
Ti	7.48%	4.55%	3.96%	7.46%

**Table 5:** Fitting parameters of the growth curve of the passive film when pH was 8.2 and 1.

	pH = 8.2	Error	pH = 1	Error
a	−0.1375	0.00822	−0.1228	0.00847
b	0.0532	0.00342	0.0519	0.00347
c	−1.241	0.01684	−1.420	0.02109

## Data Availability

The raw/processed data required to reproduce these findings cannot be shared at this time as the data is related to an ongoing study.
